# Direct lineage tracing reveals Activin-a potential for improved pancreatic homing of bone marrow mesenchymal stem cells and efficient ß-cell regeneration in vivo

**DOI:** 10.1186/s13287-020-01843-z

**Published:** 2020-07-30

**Authors:** Nidheesh Dadheech, Abhay Srivastava, Mitul Vakani, Paresh Shrimali, Ramesh Bhonde, Sarita Gupta

**Affiliations:** 1 Molecular Endocrinology and Stem Cell Research Laboratory, Department of Biochemistry, Faculty of Science, The M.S. University of Baroda, Vadodara, Gujarat India; 2 Department of Surgery, Alberta Diabetes Institute, University of Alberta, Edmonton, AB Canada; 3 Dr. D. Y. Patil Vidyapeeth, Pimpri, Pune, Maharashtra India

**Keywords:** Bone marrow mesenchymal stem cells, Activin-a, Lineage tracing, ß-cell differentiation

## Abstract

**Background:**

Despite the potential, bone marrow-derived mesenchymal stem cells (BMSCs) show limitations for beta (ß)-cell replacement therapy due to inefficient methods to deliver BMSCs into pancreatic lineage. In this study, we report TGF-ß family member protein, Activin-a potential to stimulate efficient pancreatic migration, enhanced homing and accelerated ß-cell differentiation.

**Methods:**

Lineage tracing of permanent green fluorescent protein (GFP)- tagged donor murine BMSCs transplanted either alone or in combination with Activin-a in diabetic mice displayed potential ß-cell regeneration and reversed diabetes.

**Results:**

Pancreatic histology of Activin-a treated recipient mice reflected high GFP^+^BMSC infiltration into damaged pancreas with normalized fasting blood glucose and elevated serum insulin. Whole pancreas FACS profiling of GFP^+^ cells displayed significant homing of GFP^+^BMSC with Activin-a treatment (6%) compared to BMSCs alone transplanted controls (0.5%). Within islets, approximately 5% GFP+ cells attain ß-cell signature (GFP^+^ Ins^+^) with Activin-a treatment versus controls. Further, double immunostaining for mesenchymal stem cell markers CD44^+^/GFP^+^ in infiltrated GFP^+^BMSC deciphers substantial endocrine reprogramming and ß-cell differentiation (6.4% Ins^+^/GFP^+^) within 15 days.

**Conclusion:**

Our investigation thus presents a novel pharmacological approach for stimulating direct migration and homing of therapeutic BMSCs that re-validates BMSC potential for autologous stem cell transplantation therapy in diabetes.

## Introduction

Stem cell-derived β-cells present a clear proof-of-concept for cell-based diabetes medication. After “Novacell protocol,” substantial progress has been made in generating stem cell-derived ß-cells from embryonic stem (ES) cells, induced pluripotent stem (iPS) cells, or adult progenitor cells [1–3]. BMSCs, however, show great promise for diabetes mitigation both in rodents and newly diagnosed individuals with type-1 diabetes [4–7]. Previous studies have shown that rodent BMSCs spontaneously differentiate into endocrine pancreatic cells [8–10]. The mechanism, however, remains largely unknown to state whether BMSCs can transdifferentiate into β-cells in vivo or are required to support paracrine interplay for existing β-cell growth and differentiation?

Most studies addressing the contribution of BMSCs in β-cell survival or regeneration have used transplantation of naive BMSC, while some others have used genetic tag, i.e., GFP for monitoring transplanted cells [11, 12]. An early study by Hess et al. demonstrated the blood glucose-lowering effect within a week after intravenous infusion of GFP-tagged allogenic BMSCs into STZ-induced diabetic mice [4]. The authors reported as low as 0.5% frequency of donor GFP^+^BMSC to reach the pancreas while fewer differentiate into insulin-producing cells within the host islets. In another similar study, only 1% allogeneic chimerism of repopulated BMSCs were shown to reach recipient pancreas and reverse diabetes in NOD mice [12]. Ianus et al. also showed that infused BMSCs can differentiate into insulin-producing islet cells when transplanted into lethally irradiated mice [8]. Interestingly, few other groups replicating similar studies with BMSCs reported no evidence for such an anti-diabetic effect after BMSC infusion [11, 13, 14].

The migration of BMSCs to colonize in degenerating pancreas appears to be the key for stimulating β-cell regeneration [15, 16]. Moreover, a method to stimulate pancreatic migration and trans-differentiation into β-cells limits their scope in cell therapy. It has been extensively demonstrated that the CXCR4stromal-derived factor-1 axis is crucial for BMSC migration and homing. Modulating the expression of the CXCR4 gene in BMSC could alleviate their tissue-specific homing [17, 18]. The use of chemical/biological modulators such as TGF-β family member protein Activin-a is shown to support differentiation of BMSCs [3, 19, 20]. Furthermore, Activin-a induces definitive endoderm differentiation by stimulating CXCR4 expression in ES/iPS cell-derived ß-cells and regulate migration to enhance homing [21–23].

We, therefore, used a lineage tracing approach in STZ-induced diabetic mice to demonstrate the potential of Activin-a in stimulating migration, improving pancreatic homing and efficient endogenous ß-cell differentiation.

## Material and methods

### Animals

Animal protocols were approved and performed as per Committee for the Purpose of Control and Supervision on Experiments on Animals (CPCSEA) and our Institutional Animal Ethics Committee (IAEC, MSU Baroda) (License no: 938/PO/a/06/CPCSEA) guidelines. We used male Balb/c mice, 6–8 weeks old, weighing 25–30 g and housed at 26 °C with 12 h light-dark cycle and food/water ad libitum.

### Diabetes induction and blood glucose measurement

Diabetes was induced by 5 days multiple low dose streptozotocin (STZ) injections (70 mg/kg b wt). Fasting blood glucose was monitored with Accu-Chek Glucometer.

### Isolation and purification bone marrow-derived mesenchymal stem cells

BMSCs were isolated from the tibia and femur bones of 4-week-old balb/c mice by modifying the protocols adapted from Zhu et al. and Hsiao et al. using differential trypsinization steps [24, 25]. For a more detailed protocol, please refer to supplementary methods.

### Generation of permanently labeled GFP^+^BMSC

One hundred thousand donor BMSCs were transfected with pPB-eGFP (1 μg) and pCYL43-PBase (2 μg) DNA vector (a gift from Sanger Institute, UK; for map, see supplementary figure-1) using lipofectamine 2000 (Invitrogen) in 1:3 volume ratio. Following transfection, stable GFP-expressing clones were selected on puromycin antibiotic at 300 μg/ml for the first 2 days and 900 μg/ml for the next 7 days. GFP^+^ BMSCs colonies were hand-picked using 3.2 mm clonal discs (Sigma Aldrich, USA) and FACS sorted for enriched GFP^+^ cells (see supplementary methods for cloning and purification strategy).

### Flow cytometry

Live BMSCs at p#25 or single islet cells were acquired on BD Aria-III flow cytometer for GFP^+^ cell quantification. For immunocharacterization, formalin-fixed BMSCs or islet cells were stained for MSC surface markers (CD34, CD44, CD90, CD45, CD117, Vimentin, and SMA) and endocrine differentiation antibodies (CD49b and PDX1). Cells were fixed in 4% formalin (30 min on ice), Triton-X100 permeabilized, and stained with primary labeled antibodies overnight at 4 °C for key MSCs and endocrine differentiation markers (see Table 1 for details). Data were acquired on BD Aria-III sorter using DIVA software and later analyzed with FlowJo software (FlowJo, USA).

**Table 1:** List of primary antibodies

Sr. no.	Antibody	Company and catalog no.	Isotype IgG	Mono/polyclonal Ab	Mol. weight (KDa)	Application	Dilution
1	Nestin	Sigma#N5413	Rabbit	Poly	177	Western	1:1000
2	Pdx-1	BD#554655	Mouse	Mono	40	Western/IF	1:1000/1:200
3	Neurogenin-3	Sigma #SAB1306585	Rabbit	Poly	23	Western/IF	1:1000
4	β-Actin	BD#612657	Mouse	Mono	42	Western	1:10000
5	Nestin-PE	BD#561230	Mouse	Mono	177	IF	1:100
6	Insulin	CST#4590	Rabbit	Poly	6	IF	1:100
7	Glucagon	Sigma#G 2654	Mouse	Mono	3.48	IF	1:100
8	Somatostatin	Sigma #SAB4502861	Mouse	Poly	12	IF	1:100
9	CD90.2-FITC	BD#55302	Rat	Mono	~ 20	Flowcytometry/IF	1:10/1:500
10	CD44-PE	BD#553134	Rat	Mono	82	Flowcytometry/IF	1:10/1:500
11	CD34-FITC	BD#553733	Rat	Mono		Flowcytometry	1:10
12	CD45-APC	BD#559864	Rat	Mono		Flow cytometry	1:10
13	CD117-PE	BD#553869	Rat	Mono		Flowcytometry	1:10
14	CD49b	BD#554999	Rat	Mono		Flowcytometry	1:10
15	Vimentin	Sigma#C9080	Mouse	Mono	53	IF	1:400
16	Smooth muscle actin	Sigma#F3777	Mouse	Mono	42	IF	1:250
17	C-Peptide	CST#4593	Rabbit	Mono	5	IF	1:100

### In vitro differentiation of BMSC into islet-like clusters

GFP^+^BMSCs were assessed for in vitro islet differentiation and formation of ILCC using Activin-a (20 ng/ml) as described previously [26, 27].

### Transplantation of GFP-labeled BMSCs

For lineage tracing, one million GFP^+^BMSCs were pre-incubated with Activin-a (2.5 μg/ml) for 30 min prior to the transplants and then injected intravenously into STZ-induced diabetic recipient mice, followed by daily Activin-a injections (25 μg/kg b wt) for 15 days post-transplantation. Diabetic STZ treated mice (control) did not receive donor GFP-labeled BMSC and Activin-a treatment, while a group of recipient mice received only donor GFP^+^BMSC without Activin-a treatment, served as BMSC control.

### Tissue preparation and immunohistochemistry

Pancreatic tissues from all mice at day 30 post diabetes induction were harvested, formalin-fixed, and sliced in 5 μm sections while BMSC for immunocytochemistry were fixed in 10% formalin overnight. For histology, tissue sections were deparaffinized with grading xylene and ethanol grades and rehydrated in water. Both tissues or cells were permeabilized and blocked with 4% donkey serum (Sigma Aldrich, USA) for 1 h at RT, followed by primary antibodies (see Table 1 for details) incubation overnight at 4 °C. The next day, cells were washed and labeled with secondary antibodies (see Table 2 for details) for 30 min at RT. Nuclei were marked with DAPI and mounted with Fluoromount-G (VECTASHIELD, USA). Images were captured on LSM710 confocal microscope and analyzed using Zen10 software (Carl Zeiss, USA).

**Table 2:** List of secondary antibodies

Sr. no.	Antibody	Company and catalog no.	Isotype IgG	Mono/polyclonal Ab	Application	Dilution
1	Anti-Mouse-IgG-HRP	Jackson ImmunoResearch #115-035-003	Goat	Poly	Western	1:5000
2	Anti-Rabbit-IgG-HRP	Jackson Immuno Research #111-035-003	Goat	Poly	Western	1:5000
3	Anti-Mouse-IgG-FITC	Sigma#F8771	Goat	Poly	IF	1:200
4	Anti-Rabbit-IgG-FITC	Sigma#F9887	Goat	Poly	IF	1:200
5	Anti-Mouse-IgG-CF555	Sigma#SAB4600299	Goat	Poly	IF	1:100
6	Anti-Rabbit-IgG-CF555	Sigma#SAB4600068	Goat	Poly	IF	1:100

### Protein extraction and Western blotting

FACS-sorted green cells and single-cell islet suspension from diabetic and recipient mice isolated islets were lysed in RIPA buffer (1% triton X-100, 1% sodium deoxycholate, 0.1% SDS, 0.15 mM NaCl, 0.01 M Sodium Phosphate, pH 7.2). Fifteen micrometers protein after bradford quantification was loaded on 12% SDS-page to transfer onto a nitrocellulose membrane. Membranes were blocked with 1% BSA in PBS and probed with primary antibodies (see Table 1) at 4 °C overnight. The HRP-labeled secondary antibody was then probed for 30 min at RT. Membranes were finally stained with Chemiluminescence detection reagent and images were captured on the gel documentation system (GE Healthcare). Densitometric protein expression was measured from pooled cell extracts from 3 mice in duplicates, and fold changes with SD were calculated using Fiji software.

### Serum insulin ELISA

Serum insulin from animals was measured using a mouse insulin ELISA kit (Mercodia Inc., USA).

### Statistical analysis

All statistical analysis was performed using GraphPad Prism-6 software using either two-way ANOVA or Bonferroni test for *p* value calculations with > 95% confidence. Statistics is described in legends for each figure. The number of mice transplanted is limited to *N* = 3 due to the huge cost incurred for daily Activin-a injections.

## Results

### Derivation, generation and characterization of GFP^+^BMSC

**Fig. 1:**
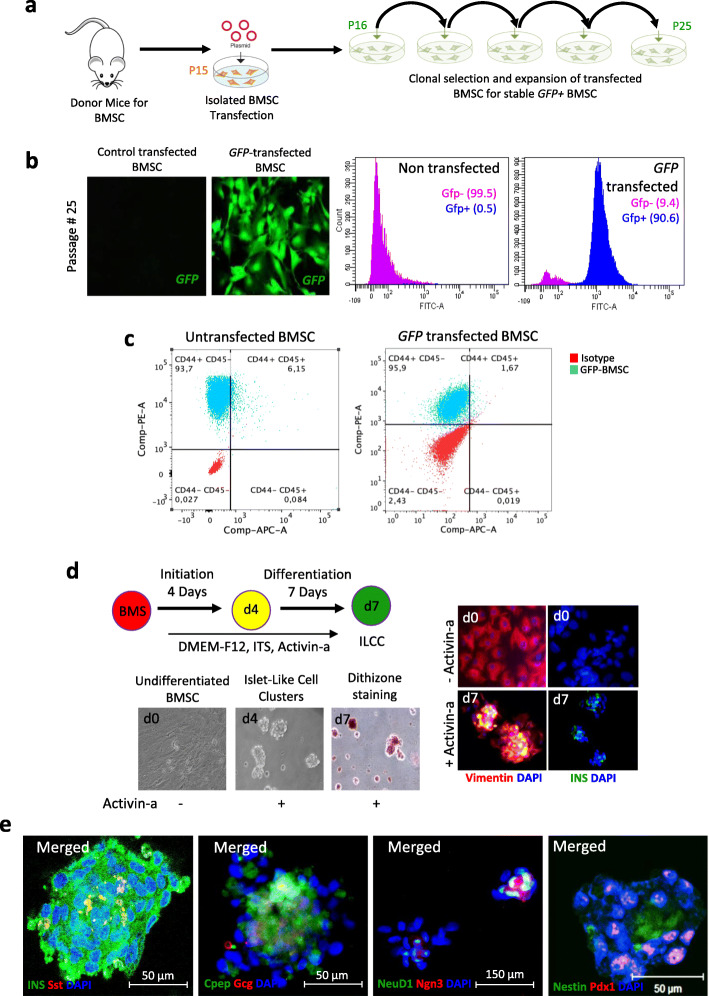
Generation of GFP tagged mouse bone marrow-derived mesenchymal stem cells and differentiation into functional pseudo-islets. **a** Schematic representation for BMSC isolation and stable GFP+ clone selection. **b** Fluorescent images of stable GFP^+^ (green) expressing BMSC and flow-cytometric quantification of sorted GFP^+^BMSC line. **c** Immunophenotyping of mesenchymal stem cell markers in GFP^+^BMSC in comparison untransfected BMSC using flow cytometry. **d** Schematic representation of islet differentiation protocol into functional islet-like cell clusters and representative microscopic images of GFP^+^BMSC at days 0, 4, and 7. Immunostaining images for vimentin (red) and insulin (green) are represented at initiation and completion of differentiation steps. **e** Immunostaining images for insulin (green) and somatostatin (red); c-peptide (green) and glucagon (red); NeuroD1 (green) and Neurog3 (red), and pdx1 (red) and Nestin (green) in differentiation islet-like clusters

We isolated BMSCs from donor mice surgically by modifying the previously described protocols from Zhu et al. and Hsiao et al. [24, 25]. A homogeneous population of BMSC without hematopoietic and macrophage contamination was achieved by differential trypsinization technique [25]. To perform lineage tracing of BMSCs, we created traceable BMSCs by permanent genomic integration of GFP using piggyback transposomal elements (Fig. 1a; Suppl. Fig-1). Transfected BMSCs show a high frequency of GFP^+^ cells with flowcytometry (90.6%). Fluorescent imaging also confirmed the presence of high GFP signals in the FACS-sorted clone (Fig. 1b, Suppl. Fig-2). We further confirmed mesenchymal markers profiling post-genetic modification and observed GFP^+^ BMSC retained the characteristics. Transfected cells displayed positive expression for CD44 (97.6%) (Fig. 1c), CD34 (88.9%), CD90 (87.5%), CD117 (18.5%), Nestin (48.9%), Vimentin (99.5%), Smooth muscle actin (89%), and pancreatic duodenal homeobox-1 (8.67%) (Suppl. fig-3), while negative expression for CD45 (1.67%) (Fig. 1c) and CD49b (0.17%) (Suppl. fig-3). These marker expressions correspond to BMSC according to the International Society of Cellular Therapy System [28]. It has been well known that human BMSCs do not express the CD34 marker, but at least in mice, there are marked differences in the CD34 expression profile. These have been discussed widely in two independent reports confirming the presence of CD34 expression in murine BMSCs [29, 30].

### In vitro ß-cell generation potential of GFP^+^BMSC

Prior to lineage tracing experiments, we examined the ß-cell differentiation potential of genetically labeled BMSCs using Activin-a growth factor, ex vivo, as previously reported [26, 31]. Genome integrated GFP^+^BMSC showed effective cell clustering at day 2 deciphered into islet-like aggregates by day 4 till day 7, similar to the non-transfected control BMSC (Suppl. fig-1b). After 7 days of differentiation, islet-like clusters were stained for dithizone staining and confirmed for the presence of vimentin expression (red) and insulin (green) (Fig. 1d). Further, the presence of insulin (green), c-peptide (green), glucagon (red), and somatostatin (red) along with other crucial pancreatic reprograming markers, nestin (red), pdx1 (green), neurog3 (red), and neuro-d1 (green), confirmed BMSC-islet differentiation (Fig. 1e).

### Model of pancreatic injury and lineage tracing of GFP + BMSC to contribute to the new ß-cell formation

To evaluate the BMSC potential for repair and restoring lost ß-cell mass, we adopted the STZ-induced diabetic model for partial ß-cell ablation and mild hyperglycemia. As per National Institutes of Health (NIH) and the Animal Models of Diabetic Complications Consortium (AMDCC), USA, the recommended blood glucose level for diabetes induction in STZ-treated mice under a non-fasting state should be > 200 mg/dl (11.1 mmol/l), whereas for a fasted animal, it should be > 150 mg/dl (8.4 mM) [32, 33]. Hence, we injected STZ at 70 mg/kg body weight for 5 days to attain glycemia > 11 mM in a fasted condition. It has been well documented that the pancreatic transcriptional reprogramming markers are only expressed at early time points for a very short duration during the β-cell regeneration process. To study BMSC-derived β-cell regeneration, it is mandated to perform lineage tracing studies early-on, post-transplantation. Hence, we performed lineage tracing on day 30.

**Fig. 2:**
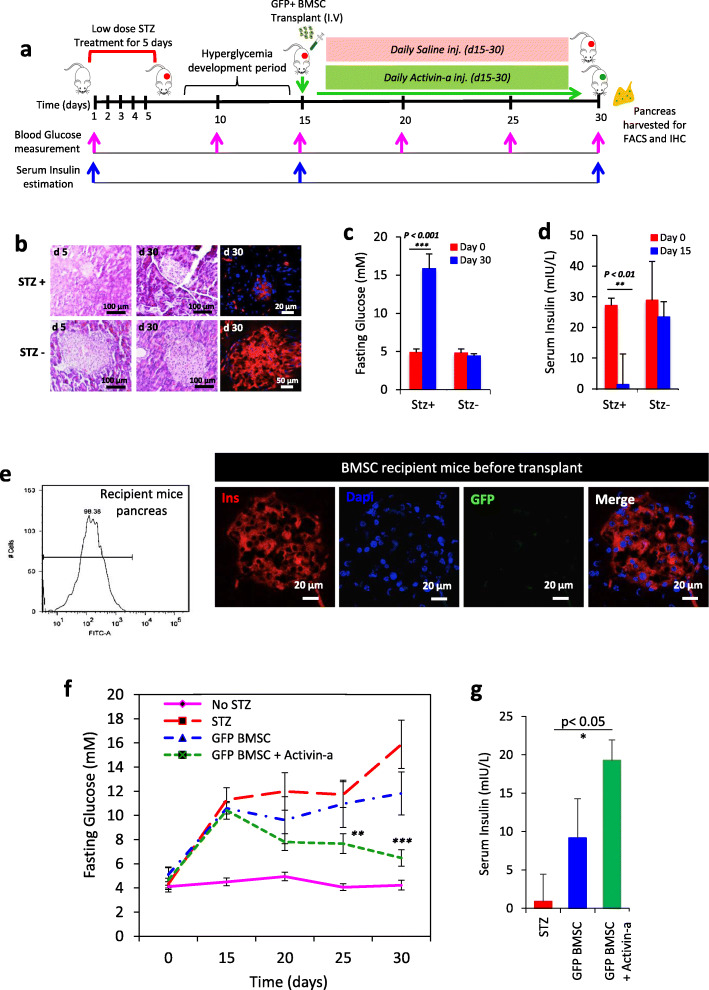
Transplantation of GFP^+^BMSC into STZ induced diabetic mice model. **a** Experimental design and timeline for the development of STZ diabetic mice and assessment of pancreatic regeneration with GFP^+^BMSC in combination with Activin-a. **b** Evidence for the establishment of diabetes and pancreatic injury after STZ injections by representative pancreatic histology (H&E) and immunostaining for insulin (red). Graphical representation of fasting **c** blood glucose and **d** serum insulin levels in control and STZ treated mice. Data represent mean ± SEM with *N* = 3 mice per group. **e** Validation of GFP expression in recipient STZ treated mice pancreas using flow cytometry and immunostaining for insulin (red). Graphs represent **f** fasting blood glucose and **g** serum insulin levels in controls and donor transgenic BMSC recipient mice. Data represent mean ± SEM with *N* = 3 mice per group. All statistical analysis was performed using Graphpad Prism software using two-way ANOVA and Bonferroni test for *p* value calculations

We designed an experimental approach to study pancreatic repair upon transplantation of donor allogeneic GFP-expressing BMSC (Fig. 2a). Fasting blood glucose more than 15 mM for 30 days and depleted serum insulin levels confirm the model establishment for ß-cell death (Fig. 2c, d) Histo-morphological assessment of pancreatic sections stained with hematoxylin and eosin (H + E) and immunohistochemistry for insulin (red) showed pancreatic injury and ß-cell damage in islets at day 30, resulting in hypoinsulinemia and hyperglycemia (Fig. 2f, g). Another set of diabetic un-transplanted representative mice (*n* = 2) was sacrificed and the pancreas was harvested solely to survey the GFP expression in pancreatic cells by flow cytometry and microscopy and found negative for GFP signals (Fig. 2e). Established hyperglycemic recipient mice were intravenously transplanted with transgenic GFP + BMSC, and blood glucose and serum insulin levels were measured. Control non-diabetic mice retained physiological glycemic control over the total duration of the study, while non-transplanted diabetic mice exhibited a hyperglycemic response after STZ injection with elevated blood glucose and severely depleted insulin levels (Fig. 2f, g). The data from mice transplanted with allogenic GFP^+^BMSC without Activin-a treatment followed a similar glycemic pattern as the diabetic controls and fails to reverse diabetes. These findings coincide with the earlier similar reports where BMSC failed to reverse hyperglycemia. Additionally, in another group of our experimental design, where mice transplanted with GFP^+^BMSC and also treated with Activin-a for 15 days, interestingly, we found a profound effect of this treatment on glucose-lowering and increased serum insulin levels after 30 days. We speculate that these results reflecting the reversal of diabetes in Activin-a treatment mice could be attributed to two possibilities: (1) GFP^+^BMSC contributes to a new ß-cell generation that resulted in increased insulin and reduced blood glucose, and (2) Activin-a treatment substantially stimulate insulin biosynthesis or release from pre-existing ß-cells. To further test this, we performed lineage tracing and surveyed GFP-expressing cells in recipient mice’s pancreas and liver. The aim is to survey for the evidence of transgenic BMSC contributing to ß-cell regeneration.

### Activin-a treatment stimulates pancreatic migration and homing of GFP^+^BMSC

**Fig. 3:**
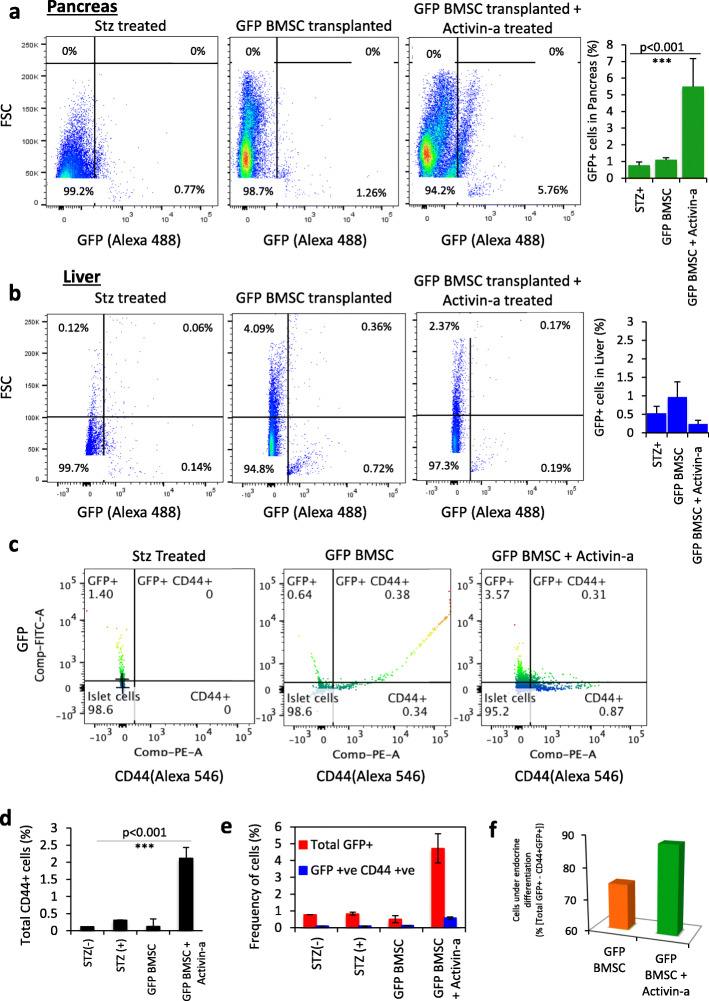
Quantification of GFP^+^BMSC in recipient mice pancreas and liver tissues. FACS analyses dot plots representing percentage population migrating to the **a** pancreas and **b** liver tissues in diabetic and donor BMSC recipient mice. Graphs present quantification of the mean frequency of GFP^+^ cells in both pancreas and liver tissues in all groups of animals. Data represent mean ± SEM with *N* = 3 mice per group. **c** Immunophenotyping of CD44 and GFP-expressing cells in FACS sorted total pancreatic cell suspension. Graphs representing quantification of **d** total CD44^+^ cells; **e** CD44^+^GFP^+^ dual population in harvested mice pancreas. **f** Graph showing quantification for the extent of endocrine differentiation in migratory donor BMSCs by reduced CD44 expression. This is calculated by subtracting CD44^+^GFP^+^ dual population from the total GFP^+^ population. Data represent mean ± SEM with *N* = 3 mice per group. All statistical analyses were performed using Graphpad Prism software using two-way ANOVA and Bonferroni test for *p* value calculations

We hypothesized that the effect on blood glucose and serum insulin levels in Activin-a treatment mice with bone marrow-derived stem cells is a result of the new ß-cell formation. To investigate this, we first examined the migration pattern and homing of GFP-expressing BMSC in diabetic control and GFP^+^BMSC transplanted mice under the influence of Activin-a treatment. Pancreas and liver tissues harvested at day 30 from all groups of animals were digested to single-cell suspension for FACS quantification of GFP^+^ cells. Whole pancreatic cells sorting from diabetic control and BMSC transplanted mice without Activin-a treatment displayed less than 1% (0.7 ± 0.44) GFP^+^ cell migrating to the pancreas, whereas BMSC recipient mice treated with Activin-a presented significantly higher GFP 6 ± 0.42% expressing cells (Fig. 3a). Subsequently, no significant migration and homing were observed into the liver in all the groups (Fig. 3b), suggesting that Activin-a could only promote efficient pancreatic lineage migration of GFP^+^ BMSC but not into the liver.

Further, to identify the specific molecular signature of pancreas migrated GFP^+^ cells, we performed FACS profiling for GFP^+^ cells with CD44 (mesenchymal marker) in the single-cell population. Both normal (0.12 ± 0.01%) and diabetic control (0.13 ± 0.01%) mice islet cells did not present CD44^+^ cells, indicating that MSCs do not considerably reside within the islets. However, untreated diabetic recipient mice displayed approximately 0.31 ± 0.21%, while Activin-a treated recipient showed a significantly high number of CD44^+^ cells (2.12 ± 0.31%), respectively, within the total cell population (Fig. 3d, Suppl. Fig-4). The fact that recipient mice received donor allogeneic BMSC, we then quantified the presence of GFP^+^ cells specifically within the islet cell population. As expected, controls and untreated recipient diabetic mice pancreata contained an extremely low number of GFP^+^ cells out of total islet population (control 0.75 ± 0.001%, diabetic control 0.83 ± 0.091%, and GFP-BMSC transplanted 0.51 ± 0.21%). Activin-a treated transplanted mice dramatically displayed a high frequency of GFP^+^ cells (4.72 ± 0.87%) within the isolated islet cell population (Fig. 3e). This implied that Activin-a treatment in recipient mice could potentially stimulate efficient migration and improved homing of transplanted BMSCs to the injured pancreas.

If the donor BMSC were to contribute to new islet cell generation with Activin-a, we hypothesize that the subset of migratory GFP^+^ cells in islets should demonstrate loss of CD44 expression without losing GFP signals. The GFP^+^CD44^−^ cells thereby present evidence of donor BMSC cell trans-differentiation into new islet cells. To do this, we FACS analyzed the dual stained (GFP/CD44) islet cells in each group. Again, no dual-stained cells in both controls were detected. A tiny fraction of undifferentiated GFP^+^CD44^+^ cells (0.13 ± 0.01%) was observed in untreated donor BMSC recipient mice. Similarly, Activin-a treated recipients demonstrated 3.67 ± 0.13% GFP^+^CD44^−^ (differentiated) and only 0.57 ± 0.07% GFP^+^CD44^+^ (undifferentiated) cells (Fig. 3c, e). The extent of differentiation of donor BMSC could be calculated by subtracting the frequency of undifferentiated cells GFP^+^CD44^+^ from the total GFP^+^ cells quantified within the islets. We observed 25% of cells (0.13 out of 0.51%) of donor GFP^+^BMSC in untreated and 88% cells (4.15% out of 4.72%) of donor GFP^+^BMSC in Activin-a treated BMSC transplanted animals undergo trans-differentiation (Fig. 3f, Suppl. Fig-5). These observations collectively indicate that despite the potential, due to the fairly low migration of transplanted donor BMSC into the injured pancreas, not enough BMSCs could deliver and transdifferentiate into new insulin-producing cells which ultimately accounts for donor BMSC failure to mitigate hyperglycemia in control BMSC alone, recipients. On the other side, Activin-a treatment in conjunction with BMSC infusion in the recipient mice demonstrated this proof-of-concept for BMSC transdifferentiation.

Although BMSC in untreated animals holds the similar potential to produce new islet cells, however, due to the fairly low migration of transplanted donor BMSC into damaged islets, not enough BMSC deliver new insulin-producing cells and ultimately fails donor BMSC to reverse hyperglycemia in non-treated animals, unlike Activin-a treated ones.

### Transplanted GFP^+^ donor BMSC gives rise to β-cells in injured pancreas revealing evidence of β-cells neogenesis with Activin-a treatment

**Fig. 4:**
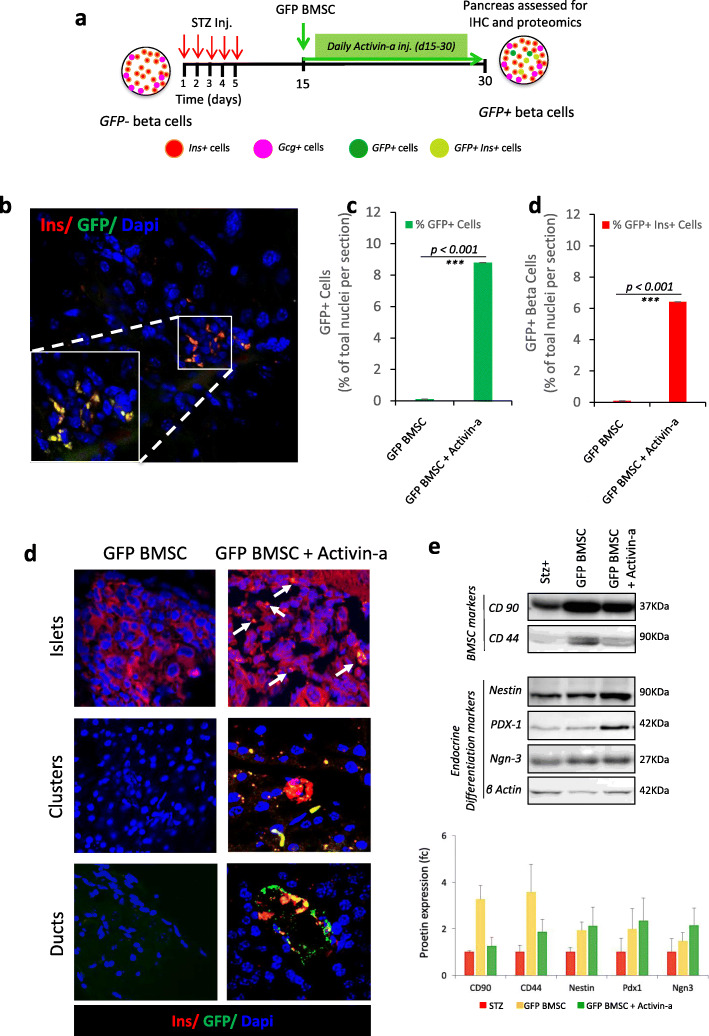
In vivo lineage tracing of transplanted GFP^+^BMSC in recipient mice pancreas. **a** Schematic representation of the experimental model to lineage trace GFP + BMSC contributing to new ß-cell generation. **b** Representative image from Activin-a treated GFP + BMSC recipient mouse pancreas showing GFP-labeled ß-cells by co-immunostained for GFP (green) with insulin (red). Nuclei were stained with Dapi (blue). The graph displays quantification of **c** total GFP+ cells and **d** GFP+ ß-cells within the islets of regenerating the pancreas. Data represent mean ± SEM with *N* = 3 mice per group. All statistical analyses were performed using Graphpad Prism software using two-way ANOVA and Bonferroni test for *p* value calculations. **e** Representative confocal microscopic images of immunostaining for insulin (red) and GFP (green) representing infiltration of transplanted GFP + BMSC in mature islets and ductal regions. Small clusters of GFP+ ß-cell present evidence for de novo BMSC-derived ß-cell formation from the transplanted BMSC. Nuclei were stained with Dapi (blue). **f** Proteomic characterization by western blotting and densitometric quantification of key pancreatic endocrine differentiation transcription factors from FACS sorted islet cells of 3 pooled representative mice pancreas, depicting evidence of new ß-cell differentiation markers. Chemiluminescence signals were exposed for 1–2 min and images were captured on the Gel Documentation system (GE Healthcare) and analyzed with ImageJ software. A single cropped area of key proteins from each condition is represented, while the graph represents densitometry quantification for each protein with standard deviation from 3 pooled mice cell extracts in duplicates

To investigate the endogenous β-cell regeneration, we compared the total number of insulin+ cells and GFP-expressing insulin cells in the pancreas. In our experimental model for lineage tracing using GFP^+^BMSC as shown in Fig. 4a, at day 30, GFP^−^Ins^+^ cells would denote endogenous β-cell regeneration while the dual-positive GFP^+^Ins^+^ cells would confirm trans-differentiation of transplanted bone marrow-derived cells. Immunohistochemistry in diabetic control mice did not display any GFP-expressing cells but reduced insulin immunopositive region depicted β-cells damaged by STZ treatment (Fig. 4b). Further, occasional scattered GFP^+^ cells were observed in the acinar region of untreated BMSC transplanted mice but devoid of insulin co-expression reflected the presence of undifferentiated BMSCs within islets. Moreover, in Activin-a-treated recipient mice, we could find a high ratio of GFP^+^ cells in acinar, ducts, and islet regions. These animals presented 8.7 ± 0.46% GFP^+^ cells, of which 6.4 ± 0.30% were GFP^+^ β-cells per section of pancreatic tissue (Fig. 4c). We recorded the GFP-expressing cells infiltrated in large-sized islets co-expressing insulin as well as small clusters or β-cell aggregates (Fig. 4d, Suppl. Fig-6). Interestingly, the entire cells in these clusters were found to be GFP positive along with insulin co-immunostaining, representing an index of β-cell neogenesis.

### Activin-a-mediated Neurogenin-3 re-activation suggests the mechanism of trans-differentiation into GFP^+^BMSC-derived β-cells

Using FACS-sorted single GFP^+^β-cells from BMSC controls and Activin-a treated BMSC recipient mice pancreas, we investigated the mechanism of new β-cell formation by protein expression. Western blot analysis for key mesenchymal stem cells (CD90 and CD44) and β-cell differentiation markers (Nestin, Pdx1, and Neurog3) suggested neuroendocrine reprogramming in GFP^+^ cells with Activin-a treatment. FACS-sorted green cells demonstrate high expression of CD90 in Activin-a-treated animals compared to STZ-treated diabetic controls. Correspondingly, CD44 remains fairly undetectable in diabetic control and Activin-a-treated groups Vs untreated BMSC recipients, suggesting lineage transformation of GFP^+^BMSC into endocrine cells with Activin-a treatment. Increased protein expression of nestin, pdx1, and neurog3 in Activin-a treated BMSC recipients provides clear evidence for pancreatic endocrine cell reprograming with sequential activation of β-cell transcription factors. Neurog3 re-activation in GFP^+^BMSC deciphers mechanism of trans-differentiation into new β-cells (Fig. 4e, Suppl. Fig-7).

## Discussion

Over two decades, several studies presented shreds of evidence for generating β-cells from BMSC [34, 35]. Despite the BMSC potential, the underlying mechanism that governs critical signals for migration and homing of BMSC remains elusive. Lack of evidence and efficacy in migration for β-cell regeneration remained questionable.

Hess et al. reported a lowering of blood glucose levels and 0.5–2% GFP^+^ cells reaching the pancreas [4]. Subsequently, others have reported no significant trans-differentiation of BMSC into insulin-producing cells, in vivo [14]. Our recent study suggests that permanently GFP-expressing BMSCs can efficiently reverse chemical-induced hyperinsulinemia and hyperglycemia. Using an endocrine cell-differentiating agent, Activin-a, we were now able to force prominent migration and colonization of GFP^+^BMSCs into the injured pancreas. We believe that pre-incubation of BMSCs with Activin-a and enhanced CXCR4 expression in infused BMSCs could potentially accelerate the pancreatic migration and triggers endocrine differentiation for β-cell trans-differentiation. Our results redefined significant migration of GFP^+^BMSC (~ 6%) into diabetic pancreas with Activin-a treatment, compared to 0.5–1% homing in BMSC transplanted/diabetic controls.

Wang et al. in 2006 reported that transplantation of GFP^+^BMSCs into neonatal mice displayed 40% cell migration into exocrine while only a few contributes to the endocrine compartment [36]. We further redefine this with Activin-a that improves the absolute homing of BMSC specifically into endocrine (islets) fraction. Flow analysis of GFP and CD44 dual markers (Fig. 3e) and insulin/GFP imaging (Fig. 4b, d) confirm this observation. Other reports raised the concern of BMSC contributing to the development of fibrosis [37–39]; however, we did not observe this. We anticipate this could be potentially an outcome of crude bone marrow population infusion (including hematopoietic cells) while we have used more enriched and characterized and BMSC populations.

The presence of GFP+ β-cells within islets and small β-cell clusters in Activin-a treated mice confers endogenous pancreatic regeneration by BMSC transdifferentiation with daily Activin-a injections. We believe this is attributed to key endocrine transcriptional reprogramming initiation with Activin-a treatment. Protein expression profiling from FACS sorted green cells display concrete evidence for new β-cell generation and reveal a new mechanism of transdifferentiation by sequential activation of β-cell differentiation markers, precisely via neurogenin-3 re-activation in migrated GFP^+^BMSCs.

Our method of endogenous pancreatic regeneration using BMSCs and differentiation growth factors like Activin-a could substantially influence a newer paradigm of cell therapy for diabetes in a wider diabetic population using GMP grade autologous BMSCs transplantation.

## Conclusion

Our study concludes Activin-a potentiation in migration, homing, and β-cell differentiation of transplanted BMSCs. This novel pharmacological approach for stimulating direct migration and homing of therapeutic BMSCs reignites the scope for autologous BMSC transplantation therapy to treat diabetes.

## Supplementary information

**Additional file 1: Supplementary Figure 1.** (a) Vector map depicting pPBGFP and pCyL43 Pbase plasmids for genomic-integrating and constitutively expressing GFP in transfected BMSC. (b) Islet differentiation stages and immunostaining representative images. **Supplementary Figure 2.** (a) Pictorial representation of BMSC clone selection strategy using flowcytometry, (b) FACS profiling, gating and sorting parameter images for positive GFP-BMSC clone, (c) Gating strategy and analysis used for population selection with doublet discrimination before BMSC surface immunophenotyping FACS quantification, and (d) representative FACS graphs for confirming cell viability and death using propidium iodide staining and fluorescent image of GFP (green) co-labelled with dapi nuclear staining. **Supplementary Figure 3.** Comparative immunophenotyping characterization of unmodified and genetically modified BMSCs with key mesenchymal, hematopoietic and pancreatic endocrine cell markers with flow-cytometry. **Supplementary Figure 4.** Comprehensive flow cytometric quantification of percentage (a) total CD44 population and; (b) GFP population and within the injured pancreas in controls non-recipients and treated BMSC recipients with and without Activin-a treatment. **Supplementary Figure 5.** Comprehensive flow cytometric quantification of percentage GFP^+^CD44^+^ expressing dual population in FACS sorted single islet cell suspension. **Supplementary Figure 6.** (a) Immunocytochemical images from islet-like structures differentiated from GFP^+^BMSC. (b) pancreatic immunohistochemical sections from GFP^+^BMSC and GFP^+^BMSC + Activin-a treated animals. **Supplementary Figure 7.** Unedited western blot images for mesenchymal stem cells and pancreatic differentiation transcription factors.

**Additional file 2:.** Supplementary Methods.

## Data Availability

All relevant data are within the paper and its Supporting Information files.

## Abbreviations

BMSC: Bone marrow-derived mesenchymal stem cells
GFP: Green fluorescent protein
ESC: Embryonic stem cells
iPSC: Induced pluripotent stem cells
STZ: Streptozotocin
FACS: Flowcytometry
