# Supplementary material for: Direct lineage tracing reveals Activin-a potential for improved pancreatic homing of bone marrow mesenchymal stem cells and efficient ß-cell regeneration in vivo
Source: Stem Cell Res Ther. 2020 Jul 30;11:327. doi: 10.1186/s13287-020-01843-z (PMC7393856; doi:10.1186/s13287-020-01843-z)
Supplement: Supplementary file 2 — Additional file 2:. Supplementary Methods. [file 13287_2020_1843_MOESM2_ESM.docx]

**Supplementary Methods.**

**BMSC isolation from femurs and tibiae:**

Mouse BMSCs were isolated from tibia and femur bones of 4-week old balb/c mice as described earlier by Zhu et al.^24, 25^. Both femur and tibia bone from hind limbs were excised, washed with PBS containing penicillin (100 IU/ml), streptomycin (100 IU/ml) (MP Biological Inc. USA), followed by a brief wash with 70% ethanol. Bone chips were again washed 3 times with PBS containing antibiotics. After washing, epiphyses at each bone end were snipped and the bone cavity was exposed. The entire lumen of the bone was flushed and scarped until pale using a 21-gauge syringe needle and cells were collected into DMEM media containing antibiotics. Post flushing the bone chip was chopped into small pieces using sterile scissors and digested with the collagenase-II enzyme at 37^0^C for 1 hour under shaking conditions. Enzyme digestion was then terminated with FBS and digested cells were collected from flow-through after discarding the bone tissues. Collected mBMSC from flushing and enzyme digested were seeded in low glucose RPMI-1640 medium supplemented with 10% Fetal Bovine Serum (FBS) for the first 24 hours to remove nonadherent cells. Attached bone marrow-derived cells were purified and expended on low plastic adherence plates adapting Hsiao et al. protocol ^25^ in DMEM high glucose medium (GIBCO) supplemented with 10% FBS, bFGF (5 ng/mL), 100 U/ml penicillin, 100 μg/ml streptomycin (GIBCO), and 12 μM  L-glutamine (GIBCO) at 5% CO2 and 37°C for 48 hrs. Fibroblastic mesenchymal morphology cells were purified in the cultures using multiple low-time trypsinization at every passage until 3-4 passage to collect low adherent cells that sequentially provided with homogeneous BMSC population. Here, confluent adherent cells post isolation were trypsinized for 60-90 sec to detach loosely attached BMSCs while more attached cells were discarded as contaminants. Only the floated detached cells were collected at this point and re-cultured into new cell culture dishes with DMEM complete media until confluent. This differential trypsinization step was repeated at every single passing until 3-4 generations for achieving a complete homogenous mBMSC population.

**GFP+ BMSC Clones isolation method and enrichment strategy**:

Following stable transfection with lipofectamine, stable GFP expressing clones were selected on puromycin antibiotic at 300µg/ml for the first 2 days and 900µg/ml for the next 7 days. GFP+ BMSC colonies (4-6 colonies) were hand-picked using 3.2mm clonal discs (Sigma Aldrich, USA) and plated on 12 well plates for clonal expansion in Dulbecco high glucose medium (DMDM; GIBCO) supplemented with 10% FBS, bFGF (5 ng/mL), 100 U/ml penicillin, 100 μg/ml streptomycin (GIBCO), and 12 μM  L-glutamine (GIBCO). Two established clones were then FACS sorted for enriched GFP+ cells using FACS Aria-III and cultured in complete DMEM media for expansion and transplantation.

**In-vitro method for islet cell differentiation using GFP+ BMSC and Activin-a:**

Murine BMSCs were allowed for differentiation using ~5 million GFP+ BMSC in a 100mm cell culture dish. The differentiation process was initiated after the cells reached <95% confluency. Plated cells were cultured in the presence of serum-free DMEM knockout media (Invitrogen, Thermo Fisher Scientific) containing Activin-a (R&D systems, USA) at 50ng/ml as differentiating agents along with insulin (5 µg/ml) transferrin (5 µg/ml) and selenite (5 ng/ml) cocktail in an eight-day differentiation protocol. The differentiation media was changed every alternate day until eight days. Islet like clusters was generated on day eight were confirmed with DTZ staining for insulin-positive ß-cells.
