# Supplementary material for: Direct lineage tracing reveals Activin-a potential for improved pancreatic homing of bone marrow mesenchymal stem cells and efficient ß-cell regeneration in vivo
Source: Stem Cell Res Ther. 2020 Jul 30;11:327. doi: 10.1186/s13287-020-01843-z (PMC7393856; doi:10.1186/s13287-020-01843-z)

Supplementary figure-1

a

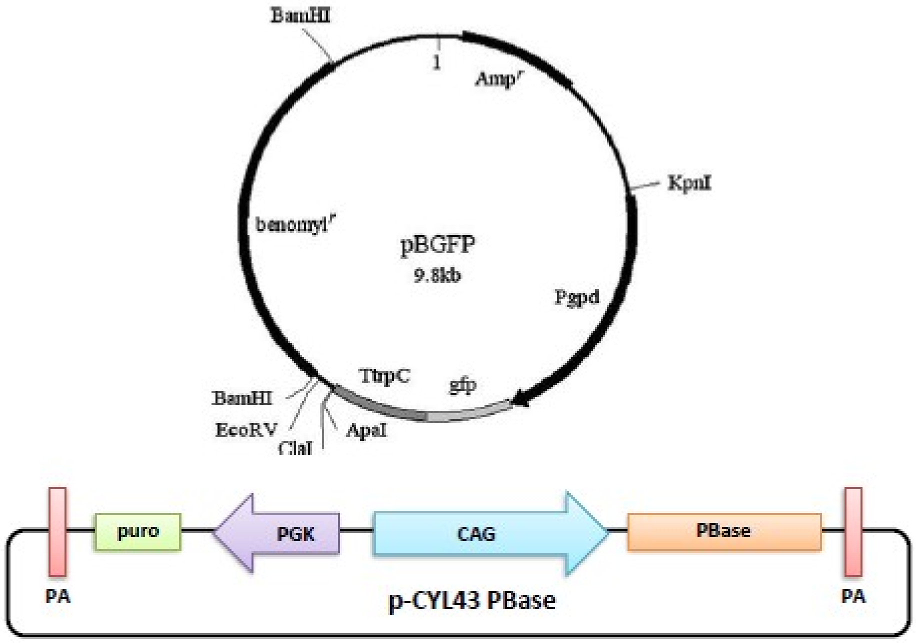

b

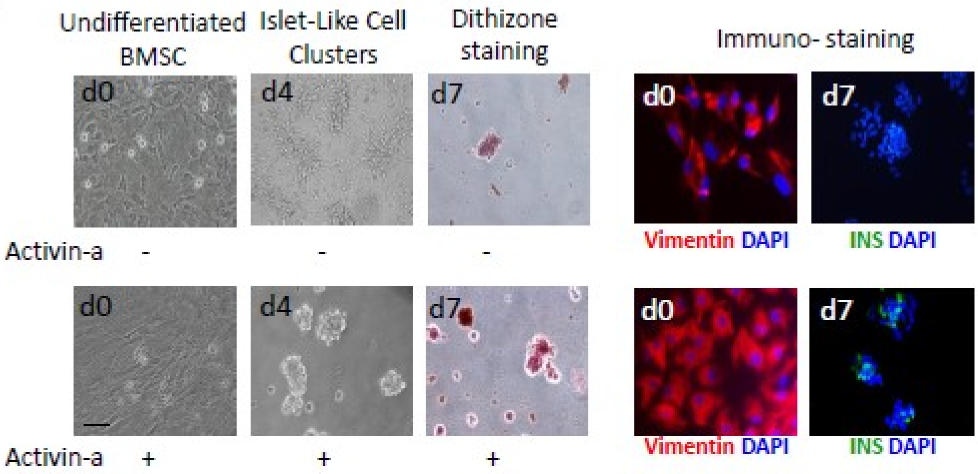

Dadheech et al., 2020

Supplementary figure-2

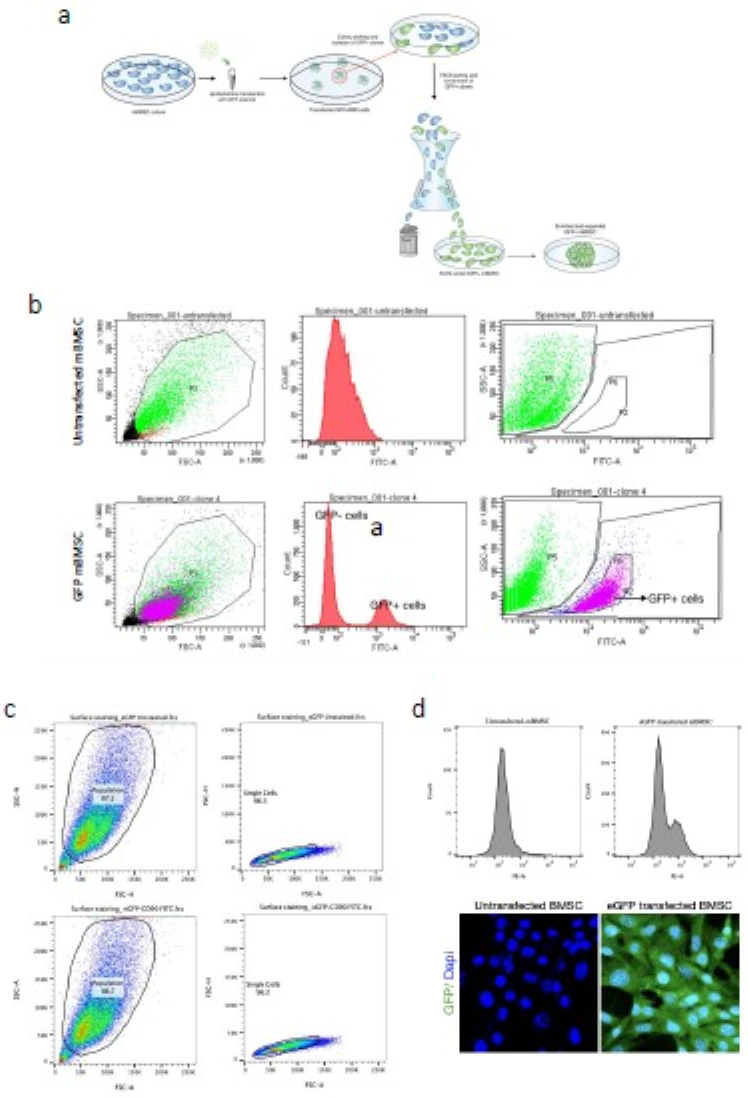

Dadheech et al., 2020

Supplementary figure-3

a

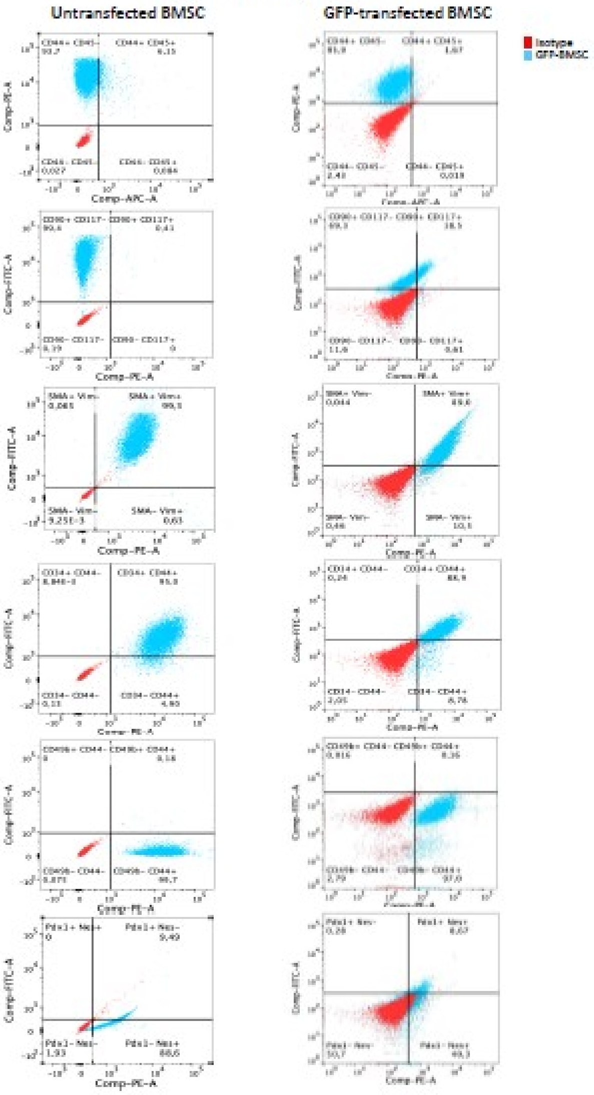

Dadheech et al., 2020

Supplementary figure-4

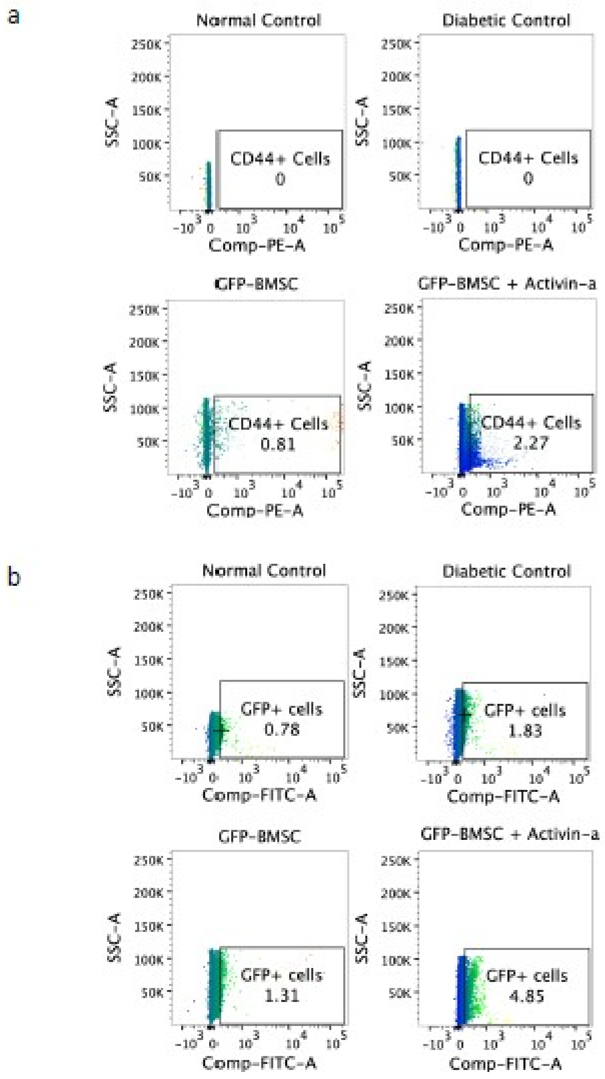

Dadheech et al., 2020

Supplementary figure-5

a

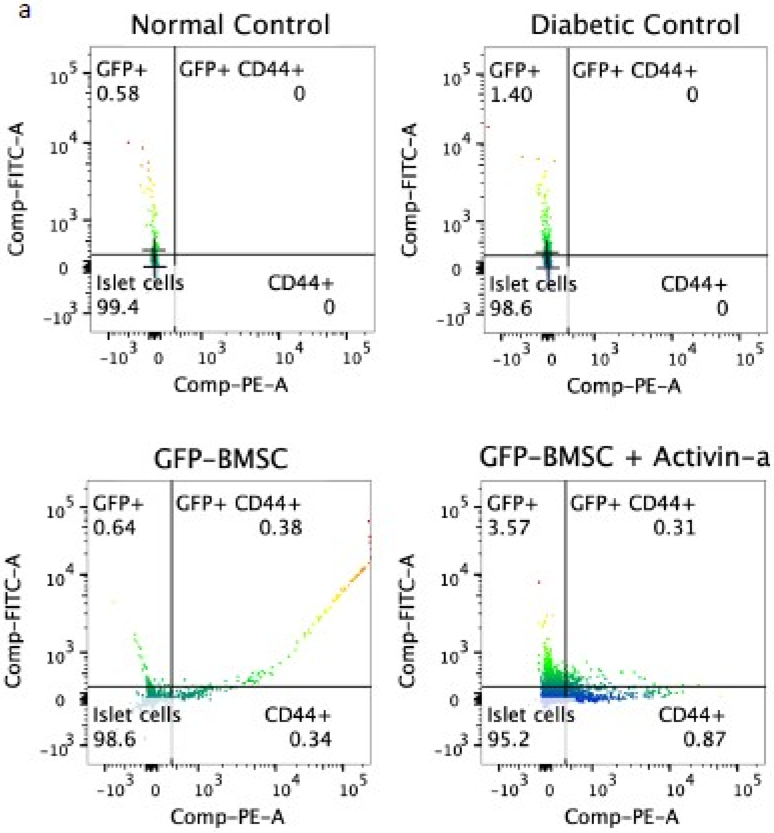

Supplementary figure-6

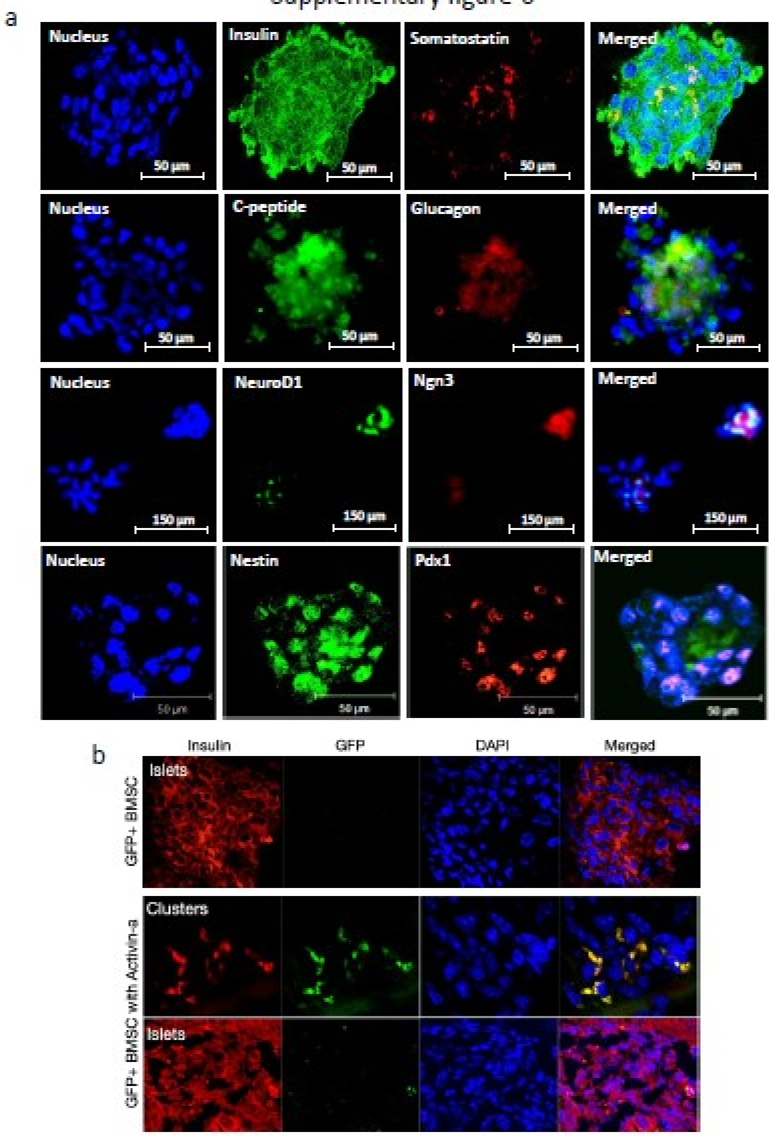

Dadheech et al., 2020

Supplementary figure-7

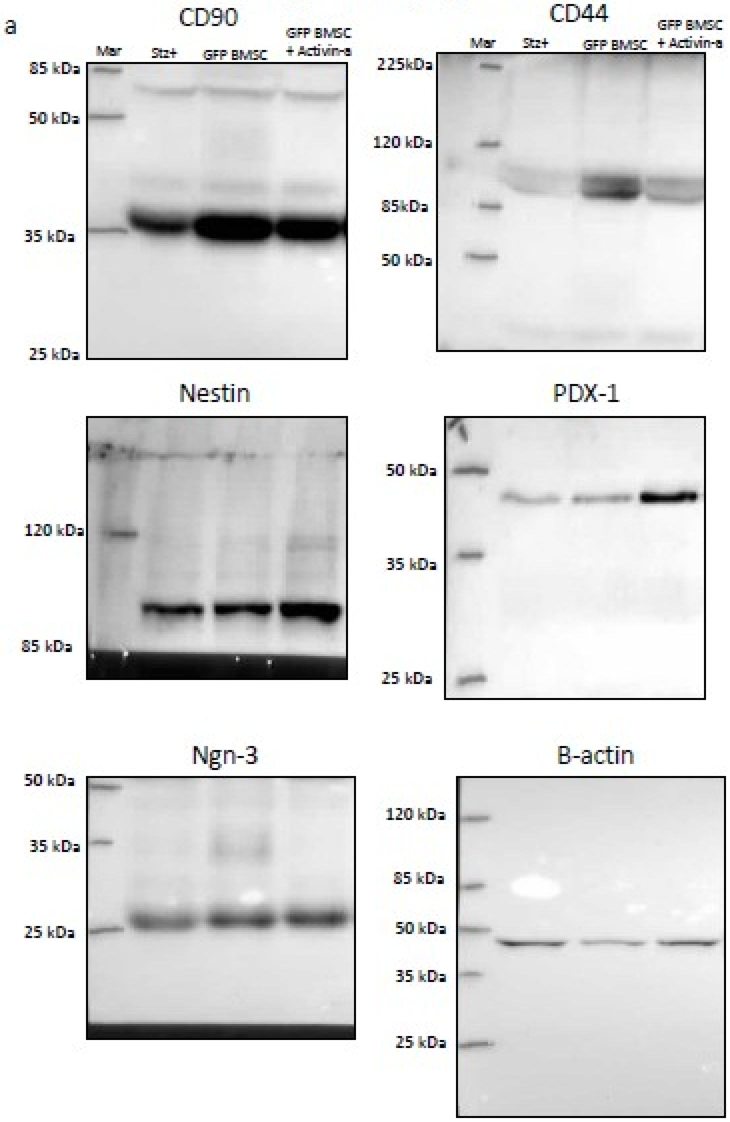

Supplement: Supplementary file 2 [file 13287_2020_1843_MOESM1_ESM.docx]
